# Genome Sequences for Multiple *Clavibacter* Strains from Different Subspecies

**DOI:** 10.1128/genomeA.00721-17

**Published:** 2017-09-21

**Authors:** Xiang (Sean) Li, Xiaoli (Kat) Yuan

**Affiliations:** Canadian Food Inspection Agency, Charlottetown Laboratory, Charlottetown, Canada

## Abstract

The Gram-positive genus *Clavibacter* harbors economically important plant pathogens infecting a variety of agricultural crops, such as potato, tomato, corn, barley, etc. Here, we report five new genome sequences, those of strains CFIA-Cs3N, CFIA-CsR14, LMG 3663^T^, LMG 7333^T^, and ATCC 33566^T^, from different subspecies of *Clavibacter michiganensis*. All these genomic data will be used for reclassification and niche-adapted feature comparisons.

## GENOME ANNOUNCEMENT

The genus *Clavibacter* was originally proposed by Davis et al. (1) to accommodate all phytopathogenic coryneform bacteria within six species of *C. michiganensis*, *C. iranicum*, *C. rathayi*, *C. toxicus*, *C. tritici*, and *C. xyli*. Subsequently, the grass-specific pathogens, *C. iranicum*, *C. rathayi*, *C. toxicus*, and *C. tritici*, were reclassified into the genus *Rathayibacter* (2), while subspecies of *C. xyli* were placed in the genus *Leifsonia* (3, 4). Currently, the genus *Clavibacter* consists of only one species, *C. michiganensis*, which is subdivided into nine subspecies, *C. michiganensis* subsp. *michiganensis* (1), *C. michiganensis* subsp. *sepedonicus* (5), *C. michiganensis* subsp. *nebraskensis* (6), *C. michiganensis* subsp. *insidiosus* (7), *C. michiganensis* subsp. *tessellarius* (5), *C. michiganensis* subsp. *phaseoli* (8), *C. michiganensis* subsp. *capsici* (9), *C. michiganensis* subsp. *californiensis*, and *C. michiganensis* subsp. *chilensis* (10).

As the causal agent of bacterial ring rot in potato tubers, the genus *Clavibacter* is regulated in North America and Europe. With more and more genomes available at GenBank, some new isolates could not be assigned correctly to any of the existing species using traditional classification methods, such as phenotypes and/or DNA-DNA hybridization values. To better define the taxonomic positions of the subspecies of *Clavibacter* on the basis of genomes (11), five *Clavibacter* genomes were sequenced. Two of these were *C. michiganensis* subsp. *sepedonicus* strains, CFIA-Cs3N and CFIA-CsR14, and three were the type strains *C. michiganensis* subsp. *insidiosus* LMG 3663, *C. michiganensis* subsp. *michiganensis* LMG 7333, and *C. michiganensis* subsp. *tessellarius* ATCC 33566; these were decoded using PacBio single-molecule real-time (SMRT) sequencing at Génome Québec (McGill University and Génome Québec Innovation Centre, Montreal, Quebec, Canada). In total, 1,959,121,498 bp, 1,975,834,871 bp, 1,904,168,446 bp, 2,128,118,724 bp, and 2,236,816,042 bp were obtained to provide approximately 536×, 537×, 458×, 516×, and 529× genome coverage for CFIA-Cs3N, CFIA-CsR14, LMG 3663, LMG 7333, and ATCC 33566, respectively. After quality checking and *de novo* assembly using the Celera assembler, the draft genome information for the strains is as follows: CFIA-Cs3N, size of 3,363,942 bp, consisting of 5 contigs, with 72.37% G+C content; CFIA-CsR14, size of 3,411,659 bp, consisting of 6 contigs, with 72.38% G+C content; LMG 3663, size of 3,387,165 bp in 3 contigs, with 72.71% G+C content; LMG 7333, size of 3,391,512 bp in 5 contigs, with 72.59% G+C content; and ATCC 33566, size of 3,318,535 bp in 2 contigs, with 73.65% G+C content. Annotation was conducted on the Rapid Annotations using Subsystems Technology (RAST) server (12) and predicted 3,133, 3,216, 3,089, 3,105, and 2,958 protein-coding genes, including 51, 51, 52, 52, and 52 noncoding RNA genes for CFIA-Cs3N, CFIA-CsR14, LMG3663, LMG7333, and ATCC 33566, respectively. In consideration of the highly specific pathogenicity of these plant pathogens on particular host plants, a total of 32 loci involved in the virulence and pathogenicity determinants for the 3 *C. michiganensis* subsp. *sepedonicus* strains were identified, in comparison with 32, 34, and 22 loci for *C. michiganensis* subsp. *insidiosus*, *C. michiganensis* subsp. *michiganensis*, and *C. michiganensis* subsp. *nebraskensis* strains, respectively.

Detailed reclassification of these genomes with others from GenBank will more accurately clarify the taxonomic status for *Clavibacter* species (11). In addition, analysis of these strains related to their niche-adapted features, including pathogenicity-related determinants, will provide detailed insight on ecology, virulence, and plant-pest interactions of these widely distributed pathogens.

### Accession number(s).

The draft genome sequences of *C. michiganensis* subsp. *sepedonicus* strains of CFIA-Cs3N and CFIA-CsR14 have been deposited in the DDBJ/EMBL/GenBank database under the accession numbers MZMM00000000 and MZMN00000000, respectively. The 3 type strains of *C. michiganensis* subsp. *insidiosus* LMG 3663, *C. michiganensis* subsp. *michiganensis* LMG 7333, and *C. michiganensis* subsp. *tessellarius* ATCC 33566 have also been deposited in the DDBJ/EMBL/GenBank database under the accession numbers MZMO00000000, MZMP00000000, and MZMQ00000000, respectively. The versions for all five strains described in this paper are the first versions.
